# Trauma-Informed Care in out-of-School time Programs: A Strategic Response to Adverse Childhood Experiences (ACEs)

**DOI:** 10.1007/s40653-025-00791-1

**Published:** 2025-11-10

**Authors:** Mary Jane Zeh, Jordan Thomas, David Gunderman, Niki Messmore

**Affiliations:** Indiana University School of Medicine, Indianapolis, IN USA

**Keywords:** ACEs, RLEs, TIC, OST programs, Childhood trauma

## Abstract

Children with multiple Adverse Childhood Experiences (ACEs) have been shown to be at greater risk of experiencing negative health and social outcomes as adults. Providing Trauma-Informed Care (TIC) to children with ACEs has been linked to better health outcomes later in life. Similarly, Out-of-School Time (OST) programs may also play an important role in supporting children who have experienced ACEs. However, few studies have examined the prevalence of ACEs among children attending OST programs specifically, or subsequently how TIC strategies can be implemented at OST programs to mitigate negative health effects. This study aimed to determine if youth who attend the studied OST Program had experienced a higher number of ACEs than the general pediatric population, and subsequently if TIC training had been implemented. An anonymous survey was developed, modeled after a template from ACEs Aware, which included ten ACEs questions (original ACEs) and nine Related Life Events questions (considered additional ACEs in this study). An optional demographics section was also included. Caregivers of children at the specified OST Program were asked to participate. Staff and volunteers were also surveyed regarding their history of training in TIC. Survey response rate was 81.3% (*n* = 52). Of the completed surveys, the mean number of total ACEs experienced per child was 4.6, the median was 3, and the mode was 2. The maximum number of ACEs experienced by a child was 14. The median number of total ACEs for the OST Program population was found to be statistically significantly different (difference = 1, *p* = 3.522e-04) than that of the general pediatric population. The median number of original ACEs experienced per child at the OST Program was also found to be statistically significantly different (difference = 1, *p* = 3.348e-05) than that of the general pediatric population. 50% of the staff and volunteers surveyed (*n* = 18, 100% response rate) had no formal TIC training. Children who attend the studied OST Program may experience on average a higher number of ACEs than the general pediatric population. Requiring training on ACEs and TIC for staff and volunteers may help better identify and respond to child behaviors linked to ACEs and improve downstream health outcomes.

## Introduction

Children who experience multiple Adverse Childhood Experiences (ACEs), such as physical abuse, sexual abuse, or substance abuse in the home, have been shown to be at greater risk for mental illness, substance abuse, and chronic health conditions as adults (Chang et al., 2019; De Venter et al., 2013; Dube et al., 2003; Felitti et al., 1998; Godoy et al., 2021; Hughes et al., 2017; LeMoult et al., 2020; Leza et al., 2021; Martinasek et al., 2021; Merrick et al., 2020; Petruccelli et al., 2019). Exposure to multiple ACEs has been shown to be “associated with higher rates of depression, tobacco use, alcoholism, illicit drug use, attempted suicide, sexually transmitted diseases, obesity, diabetes, ischemic heart disease, stroke, chronic obstructive pulmonary disease, and cancer” (Marie-Mitchell & O’Connor, 2013, p. 14). Children who experience ACEs may also exhibit different behaviors than their peers who have not experienced ACEs, such as impulsive behaviors related to maladaptive brain changes due to post-traumatic stress disorder (Lipsky et al., 2022; National Center on Afterschool and Summer Enrichment [NCASE], 2019a; Oral et al., 2016; Shin et al., 2018).

Providing Trauma-Informed Care (TIC) to children who have experienced ACEs has been linked to better downstream health outcomes (Marsac et al., 2016; Oral et al., 2016; Steen et al., 2022; Wiss & Brewerton, 2020). TIC and ACEs screening have traditionally been discussed in the context of medical practice; however, they are more recently being applied to other areas such as child care, education, juvenile justice systems, and state service providers (Graf et al., 2021; NCASE, 2019b; Stewart-Tufescu et al., 2022). For example, The National Center on Afterschool and Summer Enrichment previously hosted a webinar aimed at discussing how to mitigate the adverse effects of ACEs through state sponsored programs (Bredin et al., 2019).

Out-of-School Time (OST) programs have long been recognized as crucial for enhancing the academic achievement, nutrition, physical health, and well-being of attending youth, including academically at-risk individuals (Beets et al., 2013; Community Preventive Services Task Force, 2015; Knopf et al., 2015; Sliwa et al., 2019). OST programs may also play an important role in supporting children who have experienced ACEs by implementing TIC approaches (NCASE, 2019a; NCASE, 2019b).

Studies examining the effects of ACEs on educational, school disciplinary, and student stress outcomes with recommendations on prevention strategies to implement at school are beginning to emerge (Stewart-Tufescu et al., 2022; Pierce et al., 2022; Jackson et al., 2021; Kalmakis et al., 2020). However, few studies have examined the prevalence of ACEs among children attending OST programs specifically, or subsequently how TIC strategies can be implemented at OST programs to mitigate negative health effects later in life (Jackson et al., 2021; Kalmakis et al., 2020; Pierce et al., 2022; Stewart-Tufescu et al., 2022). This study aimed to determine whether youth who attend the studied OST Program had experienced a higher number of ACEs than the general pediatric population, thus placing them at high risk for negative downstream health effects. In conjunction, the authors examined the proportion of staff and volunteers at the OST Program that had received TIC training to determine if TIC strategies had already been implemented.

## Methods

The research focused on a specific OST Program located in a mid-sized midwestern town. The OST Program at which the research took place has two locations within the same town, both of which participated in the research. The study took place during July 2022.

An anonymous survey was developed, modeled after a template from the ACEs Aware website, which was created in partnership with the University of California San Francisco School of Medicine, Benioff Children’s Hospital Oakland, and the Center for Youth Wellness (“Screening Tools,” 2022) (Table 1). The survey included ten ACEs questions (referred to as “original ACEs”) and nine Related Life Events (RLE) questions (referred to as “additional ACEs”), two of which were for children between the ages of 12 and 18 only. All 19 questions are referred to as “total ACEs” in this study. An optional demographics section was added to the survey which included age, gender, and ethnicity. Ethnicity categories were selected using the United States Census as a guideline (Jensen et al., 2021).

**Table 1:** The survey that caregivers completed as participants of this study. Screening Tools. (2022). *ACEs Aware*. https://www.acesaware.org/learn-about-screening/screening-tools/

Questions:		
Part 1:		
1.	Has your child ever lived with a parent/caregiver who went to jail or prison?	
2.	Do you think your child has ever felt unsupported, unloved, and/or unprotected?	
3.	Has your child ever lived with a parent/caregiver who had mental health issues?	
4.	Has a parent/caregiver ever insulted, humiliated, or put down your child?	
5.	Has the child’s biological parent or any caregiver ever had, or currently has a problem with too much alcohol, street drugs, or prescription medications use?	
6.	Has your child ever lacked appropriate care by any caregiver?	
7.	Has your child ever seen or heard a parent/caregiver being screamed at, sworn at, insulted, or humiliated by another adult? OR Has your child ever seen or heard a parent/caregiver being slapped, kicked, punched, beaten up, or hurt with a weapon?	
8.	Has any adult in the household often or very often pushed, grabbed, slapped, or thrown something at your child? OR Has any adult in the household ever hit your child so hard that your child had marks or was injured? OR Has any adult in the household ever threatened your child or acted in a way that made your child afraid that they might be hurt?	
9.	Has your child ever experienced sexual abuse?	
10.	Have there ever been significant changes in the relationship status of the child’s caregiver(s)?	
Part 2:		
1.	Has your child ever seen, heard, or been a victim of violence in your neighborhood, community, or school?	
2.	Has your child experienced discrimination?	
3.	Has your child ever had problems with housing?	
4.	Have you ever worried that your child did not have enough food to eat or that the food for your child would run out before you could buy more?	
5.	Has your child ever been separated from their parent or caregiver due to foster care or immigration?	
6.	Has your child ever lived with a parent/caregiver who had a serious physical illness or disability?	
7.	Has your child ever lived with a parent or caregiver who died?	
8.	Has your child ever been detained, arrested, or incarcerated?	Children 12–18 only
9.	Has your child ever experienced verbal or physical abuse or threats from a romantic partner?	
Part 3 (Optional):		
1.	Age: ____________	
2.	Gender: □ Male □ Female □ Transgender □ Prefer not the answer	
3.	Ethnicity: □ Black/African American □ White/European □ Asian □ Hispanic/Latino □ American Indian or Alaska Native □ Native Hawaiian or Other Pacific Islander □ More than one	

Caregivers of children who attend the OST Program were approached when picking up their children and asked to participate in a voluntary survey. If a caregiver had multiple children who attended the OST Program, a survey was filled out for each child, as children in the same household still may have experienced different ACEs. A list of children whose caregivers were asked to complete the survey was kept to ensure that repeat data was not collected, as many children had different caregivers pick them up from day to day. No names of children were directly correlated with surveys in order to maintain anonymity.

The OST Program currently does not require staff or volunteers to participate in training on ACEs or TIC; thus, staff and volunteers at the OST Program were also surveyed regarding their history of outside training in TIC approaches and personal ACEs. Anonymous surveys were administered to all active staff and volunteers at the OST Program in which they answered several demographic questions, indicated their history of TIC training, and answered the same ACEs survey questions that were asked of the children’s caregivers. Participants were asked to answer ACEs questions for things experienced when they were 18 years old or younger. Each staff member or volunteer was given exactly one survey to ensure no duplicates were collected.

Data from the article by Thakur et al. (2020) was used for comparison to a general pediatric population. This randomized controlled study screened 367 children, ages 0–11 years, from the University of California San Francisco Benioff Children’s Hospital Oakland Primary Care Clinic with the same survey used in this current study (Thakur et al., 2020). Comparison was made using data from the Item-level ACEs Screen arm of the Thakur, et al. study, as the survey used in this study was an Item-level ACEs survey. Exact binomial tests were used to compare the total ACEs, total original ACEs, and individual questions with baseline values, using a significance level of 0.05. The analysis was carried out to test whether there were significant differences in the observed ACEs between the sample from the current study and the general pediatric population described in the previous research (Thakur et al., 2020).

## Results

A total of 64 surveys were distributed to caregivers of children who attend the OST Program in the midwestern town. Fifty-two surveys were completed and returned (81.3% response rate). Of the 52 completed surveys, four participants elected to not report age. Of the 48 reported ages, the average age was 9 years old. Three participants elected to not report gender identity or ethnicity. Of the participants who did report gender, an additional two marked ‘Prefer not to answer.’ Further demographics data is detailed in Table 2.

**Table 2:** Demographic distribution of participants

Age (*n* = 48)		
Average		9
Range		6–14
Gender (*n* = 49)		
Female		24 (49%)
Male		23 (47%)
Other		0 (0%)
Prefer not to answer		2 (4%)
Ethnicity (*n* = 49)		
Black/African		15 (31%)
White/European		18 (37%)
Asian		0 (0%)
Hispanic/Latino		3 (6%)
American Indian or Alaska Native		0 (0%)
Native Hawaiian or Other Pacific Islander		0 (0%)
More than one		13 (26%)

Of the completed surveys (*n* = 52), the mean number of total ACEs experienced per child was 4.6, the median was 3, and the mode was 2. Excluding questions 18 and 19, which were for ages 12–18 only, the median number of total ACEs experienced per child was still 3. The maximum number of total ACEs experienced by a child was 14 out of 19. A total of four participants reported this total ACEs number for their child. Seven children had experienced zero total ACEs, thus 86.5% of children had experienced at least one total ACE.

Looking at the ten original ACEs, the mean number experienced per child was 3.2, the median was 2, and the mode was 0. The maximum number of original ACEs experienced by a child was 9 out of 10. This number was reported by six participants. Fifteen participants reported that their child had experienced zero original ACEs. Thus, 71% of children had experienced at least one original ACE. Of the children surveyed in this study, 36.5% had experienced four or more original ACEs, compared to 13–15% in the general population found in other ACEs studies (Felitti et al., 1998; Oral et al., 2016).

Of the additional ACEs, the mean number experienced per child was 1.4, the median was 1, and the mode was 1. Excluding Questions 18 and 19, which were for ages 12–18 only, the median number of additional ACEs experienced was still 1. The maximum number of additional ACEs experienced by a child was 5 out of 9, which was reported by four participants. Seventeen participants reported that their child had experienced zero additional ACEs. Thus, 67% of children had experienced at least one additional ACE.

The final two questions were for children aged 12–18 only. The survey had ten participants state that their child was between the ages of 12 and 18. Of these ten, three answered “Yes” to the question: “Has your child ever been detained, arrested, or incarcerated?” Zero participants answered “Yes” to the question: “Has your child ever experienced verbal or physical abuse or threats from a romantic partner?”

The most common ACE experienced was Question 3 of Part 1: “Has your child ever lived with a parent/caregiver who had mental health issues?” Twenty-nine participants indicated that their child had experienced this ACE, which is 56% of the survey population. The second most common ACE was Question 10 of Part 1: “Have there ever been significant changes in the relationship status of the child’s caregiver(s)?” This question had 25 participants indicate that their child had experienced this ACE, representing 48% of the survey population (Table 3).

**Table 3:** The total number of participants who answered “Yes” per ACEs question

Question:	Number of “Yes” Answers: (*n* = 52)
Part 1:	
Question 1	20 (38%)
Question 2	16 (31%)
Question 3	29 (56%)
Question 4	11 (21%)
Question 5	17 (33%)
Question 6	14 (27%)
Question 7	23 (44%)
Question 8	8 (15%)
Question 9	4 (8%)
Question 10	25 (48%)
**Part 2**:	
Question 1	17 (33%)
Question 2	17 (33%)
Question 3	10 (19%)
Question 4	8 (15%)
Question 5	6 (12%)
Question 6	7 (13%)
Question 7	5 (10%)
Question 8	3 (30%) (*n* = 10)
Question 9	0 (0%) (*n*=10)

The median number of 3 total ACEs for the OST Program population was found to be statistically significantly different (difference = 1,*p* = 3.522e-04) than that of the general pediatric population in the referenced paper, which found a median of 2. The median number of original ACEs experienced per child at the OST Program was also found to be statistically significantly different (difference = 1,*p* = 3.348e-05) than that of the general pediatric population. The median number of additional ACEs experienced per child at the OST Program was not found to be statistically significantly different than that of the general pediatric population.

A total of 18 surveys were distributed to active OST Program staff and volunteers and 18 were returned (100% response rate). Of those that completed the survey (*n* = 18), 50% answered “No” to the question: “Have you had formal training on trauma-informed care?” However, 72% answered “Yes” to the question: “Are you comfortable and knowledgeable in working with youth who have experienced Adverse Childhood Experiences (ACEs)?”

OST program staff and volunteers were also surveyed about their personal experience with the original ACEs. The mean number of original ACEs experienced per staff member or volunteer was 1.7, the median was 0.5, and the mode was 0. The two most experienced original ACEs were Question 3 of Part 1 (“Have you ever lived with a parent/caregiver who had mental health issues?”) and Question 7 of Part 1 (“Have you ever seen or heard a parent/caregiver being screamed at, sworn at, insulted, or humiliated by another adult? OR Have you ever seen or heard a parent/caregiver being slapped, kicked, punched, beaten up, or hurt with a weapon?”). Both questions had five participants report experiencing them, which is 28% of the survey population (Table 4).

**Table 4:** The total number of staff/ volunteers who answered “Yes” per original ACEs question

Question:	Number of “Yes” Answers: (*n* = 18)
Part 1:	
Question 1	2 (11%)
Question 2	2 (11%)
Question 3	5 (28%)
Question 4	4 (22%)
Question 5	4 (22%)
Question 6	1 (6%)
Question 7	5 (28%)
Question 8	3 (17%)
Question 9	1 (6%)
Question 10	4 (22%)

## Discussion

The results of this study indicate that children attending the OST Program experience a higher number of ACEs than the general pediatric population. This would indicate that youth who attend this OST program may be at higher risk for mental illness, substance abuse, and chronic health conditions as adults, and demonstrates the need for implementation of TIC training and approaches at the OST program to mitigate these outcomes.

Similar studies have also shown that experiencing four or more original ACEs “increases the rate of smoking (2.2 times), alcoholism (7.4 times), substance abuse (4.2 times), intravenous illicit substance abuse (11.3 times), severe obesity (1.6 times), and sexual intercourse with 50 or more partners (3.2 times)” (Oral et al., 2016, p. 229). The results of this study indicate that children who attend the OST Program are more likely to experience four or more original ACEs than the general population, thus may be at a higher risk of experiencing negative health and social outcomes.

These results pose a unique intervention opportunity. This research demonstrates that OST programs may be high-yield targets for adverse outcome prevention and/or intervention strategies. Implementing required TIC training for staff and volunteers may be a pivotal adverse health outcomes prevention strategy, as only half of the surveyed staff and volunteers reported having TIC training. OST programs which have implemented TIC practices and programs have been associated with an increase in positive self-perceptions and decrease in problem behaviors, as they provide critical OST engagement with high-risk youth, where the effects of ACEs may be able to be identified and addressed to mitigate negative sequalae that arise from these traumatic experiences (Gleason et al., 2016; NCASE, 2019a).

Currently, similar OST programs in California run trauma-informed learning programs, including staff training and partnerships with local mental health professionals and social workers (Bedy, 2021). The state of Tennessee runs an ACEs initiative called Building Strong Brains, in which public and private sector partnerships are utilized to train community members in TIC (Bredin et al., 2019). Furthermore, the Substance Abuse and Mental Health Services Administration runs the National Center for Trauma-Informed Care, which provides support to organizations looking to become trauma-informed (Oral et al., 2016). Additional interventions include youth leadership programs, connecting children who have experienced ACEs to therapy, and providing grant funding for TIC training of OST staff, parents, and caregivers (Felter et al., 2023; Fondren et al., 2020; NCASE, 2019a).

While innovative programs have begun to be implemented in various states, this research further emphasizes that TIC is not yet a widespread strategy at OST programs. Programs such as The National Center on Afterschool and Summer Enrichment continue to offer examples of tangible solutions, however, OST programs must be intentional about incorporating these programs (Bredin et al., 2019). Furthermore, widespread application of TIC would undoubtedly have an increased likelihood of success with increased state or federal funding, as demonstrated by the programs discussed in California and Tennessee (Bedy, 2021; Bredin et al., 2019).

It would be remiss to conduct research about ACEs and TIC without engaging in a discussion regarding the ethical dilemmas these topics bring. Conducting an ACEs survey is a sensitive undertaking, as ACEs questions pose the risk of re-traumatization should children themselves be the primary population surveyed. Conversely, caregivers may feel targeted, inadequate, or defensive should they be the primary population surveyed. In the context of this research, the authors felt it best to choose caregivers as the primary population survey, so as not to expose the children to re-traumatization. Authors engaged in discussion with caregivers prior to providing the survey questions in order to provide sufficient context for the questions. It was emphasized that this survey was not being conducted to single out any specific child’s living situation, but rather to learn about the collective background of the children that attended this specific OST Program with the intent of better serving the children. The anonymity of the survey was reiterated, and all caregivers were provided the contact information of the authors should they have further questions. The authors were largely met with positive remarks from caregivers when conducting this survey, with caregivers voicing their gratitude that leadership at the OST Program were attempting to better inform themselves on the children’s needs.

The authors of this research chose to utilize survey questions modeled after a template from the ACEs Aware website (“Screening Tools,” 2022) (Table 1). This survey format was chosen due to its thorough development by Kaiser Permanente and the Centers for Disease Control and Prevention, as well as its use in previous research for comparison. While this survey format is nationally recognized, it is not perfect. As discussed above, the primary survey population for this research was caregivers. This poses a limitation, as caregivers may not be fully aware of trauma that their children experience. Additionally, caregivers may not be fully truthful when answering survey questions, or may not be fully aware of how their own behavior may be contributing to the trauma experiences of their children. Thus, the answers to this survey must be taken as subjective, rather than thoroughly objective answers as a survey would ideally have.

Furthermore, the questions posed in this survey have a largely negative connotation. Research has indicated that positive experiences may serve as a buffer for, or even counteract, the negative impacts of ACEs (Crandall et al., 2019; Perry, 2018). Including questions about strong relationships between the child and an adult, feelings of support or love, or other positive questions may provide further insight into the child’s true risk for adverse outcomes. Future studies should thus consider the inclusion of questions with a positive connotation into the survey.

## Conclusion

Children who attend the studied OST Program may experience on average a higher number of ACEs than the general pediatric population. Requiring training on ACEs and TIC for all staff and volunteers may help them better identify and respond to child behaviors linked to experiencing ACEs. Helping children feel safe and empowered through TIC provided at OST programs may have a positive impact on their overall physical and social health as a child, as well as in adulthood.

More research needs to be done on the average number of ACEs experienced by children attending OST programs, including surveying additional OST programs in a variety of locations. The inclusion of questions with a positive connotation should also be considered. Additional research on the impact of TIC training of staff and volunteers should also be a priority. A limitation of this study includes using caregivers as the primary survey participants, as they may not be fully aware of things their children experience. Additional limitations include relatively small sample size, surveying of a single OST Program, and the inclusion of siblings and children living in the same household, whereas the Thakur et al. (2020) study excluded siblings.

It must also be noted that the authors intentionally discussed the results of this survey in the context of identifying OST programs as “high-yield targets” of TIC. This does not mean that every child who attends an OST program has experienced more ACEs than the average population, a dangerous assumption that could lead to stigmatization.

## Declarations

This work was made possible by AmeriCorps and the Indiana University School of Medicine AmeriCorps program, Hoosier Public Health Corps, and supported by the Boys and Girls Club.
